# Using symbolic machine learning to assess and model substance transport and decay in water distribution networks

**DOI:** 10.1038/s41598-024-53746-1

**Published:** 2024-02-08

**Authors:** Daniele Biagio Laucelli, Laura Enríquez, Juan Saldarriaga, Orazio Giustolisi

**Affiliations:** 1 Polytechnic University of Bari, Via Orabona, 4, Bari, Italy; 2 Universidad de los Andes, Carrera 1 Este, 19 – 40, Bogotá, Colombia; 3 Polytechnic University of Bari, Via Orabona, 4, Bari, Italy

**Keywords:** Engineering, Civil engineering

## Abstract

Drinking water infrastructures are systems of pipes which are generally networked. They play a crucial role in transporting and delivering clean water to people. The water quality analysis refers to the evaluation of the advective diffusion of any substance in drinking water infrastructures from source nodes. Such substances could be a contamination for the system or planned for the disinfection, e.g., chlorine. The water quality analysis is performed by integrating the differential equation in the pipes network domain using the kinetics of the substance decay and the Lagrangian scheme. The kinetics can be formulated using a specific reaction order depending on the substance characteristics. The basis for the integration is the pipes velocity field calculated by means of hydraulic analysis. The aim of the present work is to discover the intrinsic mechanism of the substance transport in drinking water infrastructures, i.e., their pipes network domain, using the symbolic machine learning, named Evolutionary Polynomial Regression, which provides “synthetic” models (symbolic formulas) from data. We demonstrated, using one real network and two test networks, that the concentration at each node of the network can be predicted using the travel time along the shortest path(s) between the source and each node. Additionally, the formula models provided by symbolic machine learning allowed discovering that a unique formula based on kinetic reaction model structure allows predicting the residual substance concentration at each node, given the source node concentration, surrogating with a good accuracy the integration of the differential equations.

## Introduction

Drinking water infrastructures (DWIs) are assets playing a crucial role in transporting and delivering clean water to people, thus providing an essential service to billions of people worldwide. They are required to efficiently supply water with adequate quality for people health, thereby ensuring equitable access to water for all citizens.

DWIs encompass both Water Transmission (WTS) and Water Distribution (WDS) Systems. The former, generally characterized by long pipelines with large diameters, have few or no connections to the consumers property, while the latter are characterized by shorter pipelines with smaller diameters and several connections to properties to feed consumers. WTSs facilitate the transfer of water volumes to consumption centres (towns or cities). WDSs, also called Water Distribution Networks (WDNs), because of their networked structure, transfer water to end consumers. Therefore, the issue of water quality with respect to the contamination and disinfection for people health is mainly related to WDNs.

Water quality modelling in WDNs deals with the evaluation of water age, water trace and transport of the reactant substances considering the decay due to chemical reactions. A substance concentration over time is assumed entering from a source node and the calculation aims at determining the concentration of such substance in each node of the network allowing the assessment of the concentration to consumers. To the purpose, assuming the advective diffusion, the differential equations based on a specific kinetic model need to be integrated in the pipes network domain to calculate the concentration of the substance over time to any node. Therefore, the calculation is based on the pipes velocity field deriving from the hydraulic analysis of the system. The hydraulic analysis is based on a steady-state assumption, i.e. neglecting the unsteady flow. Therefore, assuming a time interval mainly for consumers’ demand stationarity, the basis of the hydraulic analysis is the daily scale (i.e., one or more operative cycles) and each snapshot of the hydraulic simulation refers to a consumers’ demand varying over time, following a specific demand pattern.

The integration of the differential equations is generally performed using the Lagrangian scheme:

- A water quality time step for analysis is assumed, e.g. from 1 to 5 min. The time step of the analysis is generally lower than the hydraulic time step, e.g., one hour, therefore the velocity in each pipe changes with the hydraulic time step.
- The pipes in the network are divided in parcels whose number depends on the specific velocity, i.e. the travel time. Thus, the number of the parcels changes over the hydraulic time step and there is the issue of the maximum number of parcels versus accuracy. In fact, the water quality modelling is here performed without limiting the number of parcels, see^1^ for more details.
- The kinetic reaction model is applied to the substance at every water quality time step in each parcel, if any, and they are moved forward in the direction of flow.
- The substance in the last parcel of each pipe, in the flow direction, is moved at the node and mixed assuming the instantaneous complete mixing.
- The concentration at the node is the initial condition, i.e., the concentration of the first parcel of each pipe in the direction of flow.
- Once the pipes velocity changes, the number of parcels needs to be changed and this fact asks for a mapping scheme from a lower to a greater number of parcels and *viceversa*. A correct mapping is a relevant issue for accuracy as reported in^1^.

Note that for water quality assessment the correctness of the pipes velocity field is mandatory. Therefore, we here used advanced hydraulic modelling; it allows the calculation of pressure dependent leakages at pipe level which are outflows influencing the pipes velocity field in real networks, that are generally deteriorated. The water quality calculation is time consuming because the Lagrangian scheme involves many pipes and needs to be iteratively performed assuming more than one time the same operative cycle^1^. In addition, the calibration of the kinetic model parameters and the identification of the order are relevant issues.

Therefore, the aim of this work is to investigate the mechanism of substance transport and decay in WDNs building a dataset for machine learning by means of water quality calculation, applied to a specific WDN, varying the kinetic model order, the concentration at the source node and the reaction rate parameter.

The aim is then to understand the relevant mechanism of the concentration decay from a source node to any node of the pipes network domain. For this purpose, symbolic machine learning is used to develop a unique “synthetic” model (symbolic formula) to predict the concentration at each node of the network depending on the concentration at a source node. The selected machine learning strategy was Evolutionary Polynomial Regression (EPR)^2,3^, because it allows providing symbolic formulas for models from the dataset of the water quality calculation to ascertain the mechanism and identify the kinetic order.

Thus, it is useful for the reader to report EPR in the context of machine learning. EPR is founded on the idea of evolutionary optimization integrated with machine learning. John Koza was the pioneer who developed the paradigm of *genetic programming*, showing in a book^4^ the possibility of creating machines that program themselves to solve problems postulated by humans. *Genetic programming* integrates *machine learning*, in a wider sense with respect to the original studies, with *evolutionary optimization* in an original way. *Symbolic modelling* is a specific application of Koza's paradigm to obtain models by means of the integration of *machine learning* and, e.g. *genetic algorithms*^5,6^ as an evolutionary optimization technique, in form of *symbolic formulas for models* that can be evaluated as such by the expert.

EPR is then a *genetic programming* strategy coding in an original way the problem to obtain formulas for models and involving multi-objective analysis through *genetic algorithm* (EPR-MOGA, see^3^). In fact, EPR-MOGA returns the so-called *Pareto front of optimal* or *efficient solutions*^7^ considering model complexity versus fitting to data. The EPR paradigm is opposed to other machine learning techniques such as *artificial neural networks*, which are general mathematical structures characterized by the “universal” ability to interpolate data, but, for this reason, not suitable for the interpretation of the results with respect to the physical knowledge of the expert about the modeled cause-effect phenomenon.

In brief, EPR-MOGA is a strategy to search for symbolic formulas for models belonging to a domain prior assumed by experts in an organized way. It returns the model formulas with the best trade-off of complexity (parsimony) versus fitting to data, and the expert takes the decision on the best model looking at the whole Pareto front and their symbolic structure (i.e., understandability), also considering the added value of the increasing complexity versus fitting to data.

EPR findings were validated by means of a different (i.e., unseen) dataset from that used to generate the symbolic formulas. For this purpose, a new water quality modelling run, using variable reaction rate parameter for each pipe, was performed. Such validation phase was important to discuss the meaning of the parameters to be used in the symbolic formulas obtained by machine learning. In fact, the physical meaning of the parameters of the “synthetic” model, encapsulating the substance decay mechanism in the pipes network domain, could be useful to address the issue of calibrating the kinetic model from real data, given that the pipes in the shortest path mainly determine the decay. The results are evaluated on three WDNs: two simple test networks and one real WDN.

Without impairing the generality of the procedure, the reaction rate parameters consistent with chlorine were used for water quality modelling, because chlorine is extensively used as disinfection substance in WDNs. In this sense, it is desirable to keep a certain level of chlorine residual at each node of the network^8^ based on the substance decay and dose in the source node. On the other hand, the reaction of chlorine with organic and inorganic compounds, that are naturally found in water, leads to the formation of disinfection by-products (DBPs), that have been related to serious side effects, such as cancer and congenital malformation^9^. From this point of view, chlorine dosing should be reduced to keep low DBPs levels. Hence, monitoring the chlorine residuals throughout a WDN becomes a fundamental task to reach a trade-off between these conflicting objectives.

The chlorine reactions have been classically modelled using first order kinetics^10^, but several second order models have also been proposed^11,12^. The second order model with a single reactant species has been applied by authors such as^13–15^. Other second order models with multiple competing species have also been introduced^12,16^. According to^11^, the initial disinfection that occurs in the water treatment plant may be more appropriately modelled with a second order model since a rapid initial loss takes place. Conversely, when the disinfected waters reach the distribution system, the decay rates are reduced and both first and second order kinetics may apply.

From the perspective of calibration, the estimation of the parameters of chlorine decay models is generally done using a heuristic optimization (e.g., Genetic Algorithms, Particle Swarm Optimization) to find a feasible solution^17–19^. The evaluation of each solution requires to run a simulation algorithm to estimate the chlorine concentrations over time throughout the WDN. Although approximate analytical solutions have been proposed for chlorine decay models^20^, which facilitates the calibration procedure, a transport algorithm is still necessary to compute chlorine concentrations throughout the network. Consequently, calibrating chlorine decay models is generally computationally expensive, which has limited the use of chlorine decay models for modelling purposes^15^.

Given the challenges that arise from modelling and calibrating chlorine transport, multiple authors have used data-modelling (today also named machine-learning or artificial intelligence) to predict and model chlorine decay in WDNs based on field measurements of hydraulic and water quality data. Some examples of this type of studies are^21–24^. In this context, the use of data-modelling has also been focused on finding the most influential parameters on chlorine decay considering field observations in WDNs^25^. On the other hand, some authors have used machine-learning to encapsulate the tendency of chlorine transport differential equations throughout a WDN. Particularly, *artificial neural networks* have been the predominant approach applied for this purpose. May et al.^25^ demonstrated the accuracy of a *general regression neural network* to model the chlorine concentration at a single node of a real-scale WDN using synthetic data that followed the first-order decay model. Furthermore^26^, proved that a single *artificial neural network* can calculate the chlorine concentration of a multicomponent reaction transport model at multiple nodes of different WDNs.

The proposed methodology, aimed at tackling the complexity of modelling and calibrating substance (e.g., chlorine) transport in WDNs, includes the following novelties:

- The improvement in the accuracy of hydraulic and water quality calculations with an advanced modelling approach that considers pressure-dependent leakages at single pipe level, and a Lagrange scheme with unlimited number of parcels, see^1^ for further details.
- The use of symbolic machine learning to understand the mechanism of substance transport and decay with first or second order kinetics.
- The development of a unique formula to predict the substance concentration in all the nodes as a function of the reaction rate parameter and the structure of the kinetic equation, with the possibility of surrogating the computation of water age with the travel time in the shortest path(s).
- The capacity of distinguishing between first and second order kinetics through data with a machine learning approach.

Regarding novelty (a), other studies that have used simulated data to train chlorine data-based models have not included leakages^25,26^. This is a relevant consideration since leakages are associated with a high volume of water loss^27^, which may have a significant impact on velocity fields and chlorine patterns^1,22^. Therefore, given that the hydraulic and water quality simulated data has a high accuracy level, the results of the EPR strategy are not impaired by estimation errors.

Regarding novelty (b), the selected machine learning strategy is EPR-MOGA^2,3^, because it allows providing symbolic formula for models from the dataset of the water quality calculation. As introduced above, it can return model formulas with the best trade-off between complexity and accuracy to data, so that the analyst can take the decision on the best model looking at the Pareto front and their symbolic/explicit (understandable) structure.

In this way, the choice of a single formula model that explains substance behaviour (e.g., chlorine) and its transport mechanism in the pipes network domain can have multiple potential applications for modelling, calibration, and optimization purposes. For example, a good estimation of chlorine concentration throughout a WDN can be easily and rapidly calculated with this approach given the values of the input variables without solving differential equations, which is also useful to have a less time-intensive algorithm for optimization purposes (e.g., calibration).

More details about EPR-MOGA are in the following paragraph 2.4.

Regarding the novelty (c), the calculation of nodal water age, i.e., the time the water travels from the source node to each node of the network, is computationally intensive. Researchers attempt to calculate the water age using complex network theory and shortest paths^28^ limiting the proof to branched network without devices. The present work introduces the alternative approach of surrogating the water age by means of shortest paths based on the velocity field. The accuracy of such approach can be prior evaluated for the studied WDN using the presented framework. However, further studies can allow predicting the accuracy based on the characteristic of the pipes network domain of the hydraulic system such the average nodal degree or the density of loops.

## Methodology

As previously reported, this work aims to investigate the mechanism of substance transport and decay in WDNs building a dataset for machine learning by means of water quality analysis. This is performed by integrating the differential equation in the pipes network domain using the kinetics of the substance decay and the Lagrangian scheme. The basis for the integration is the pipes velocity field calculated by means of hydraulic analysis.

To this purpose, advanced hydraulic modelling, involving pressure dependent leakage model at pipe level, is the methodology to compute the pipes water velocity^1^.

Then, the first or second order kinetic reactions was used for substance transport, assuming the reaction rate parameters consistent with chlorine, without impairing the generality of the results.

EPR^2^ was the used symbolic machine learning methodology. It provided nodal prediction models of the substance decay which are understandable formulas models to study the mechanism of substance transport and decay in WDNs, i.e. the pipes network domain.

### Advanced hydraulic modelling

The basis of the water quality computation was the field of water velocities in pipes, which was obtained from the hydraulic analysis. The mathematical representation of a hydraulic network includes *n*_*p*_ pipes with unknown flow rates, *n*_*n*_ nodes with unknown heads and *n*_*0*_ nodes with known heads. The hydraulic calculation solves the following non-linear system of equations, based on the principles of energy and mass balance^29^:

1 $$\begin{aligned} & {\mathbf{A}}_{pp} {\mathbf{Q}}_{p} \left( t \right) + {\mathbf{A}}_{pn} {\mathbf{H}}_{n} \left( t \right) = - {\mathbf{A}}_{p0} {\mathbf{H}}_{0} \left( t \right) \\ & {\mathbf{A}}_{np} {\mathbf{Q}}_{p} \left( t \right) - \frac{{{\mathbf{V}}_{n} \left( {{\mathbf{H}}_{n} \left( t \right)} \right)}}{\Delta t} = {\mathbf{0}}_{n} \\ \end{aligned}$$

where **A**_*pp*_ = [*n*_*p*_*,n*_*p*_] is a diagonal matrix with elements based on the pipes’ resistance; **Q**_*p*_ = [*n*_*p,*_1] is a column vector of unknown pipe flow rates; **H**_*n*_ = [*n*_*n,*_1] is a column vector of unknown nodal heads; **H**_*0*_ = [*n*_*0,*_1] is a column vector of known nodal heads; **0**_*n*_ = [*n*_*n,*_1] is a column vector of null values; **V**_*n*_ = [*n*_*n,*_1] is a column vector of water volume withdrawals in the nodes; Δ*t* is the hydraulic timestep;** A**_*pn*_ = **A**_*np*_^*T*^ and **A**_*p0*_ are the topological incidence sub-matrices of size [*n*_*p*_*,n*_*n*_] and [*n*_*p*_*,n*_*0*_], respectively, derived from the general topological matrix **Ā**_*pn*_ = [**A**_*pn*_ ¦ **A**_*p0*_] of size [*n*_*p*_*,n*_*n*_ + *n*_*0*_].

The hydraulic analysis for the case studies was carried out with the WDNetXL platform using the Darcy-Weisbach head loss equation. This approach is based on a realistic background leakages model that represents them as outflows at pipe level that depend on the average pressure at each pipe and its deterioration^1,29^. The hydraulic calculation incorporated a pressure-driven analysis, in which the background leakages and the water demand associated to consumers depend on the pressure throughout the system^29^. Then, the advanced hydraulic modelling allows for an accurate estimation of the water flowing in the pipes of the network and the velocity field for any water quality analysis.

### Order reaction and kinetics model

The classic equation for calculating the first-order concentration decay rate of a reactant [*A*] is:

2 $$r = \frac{d\left[ A \right]}{{dt}} = - K\left[ A \right]$$

where the decay rate *r* is formulated following first order kinetics and *K* is a positive constant called reaction rate parameter, that represents the initial rate of the reaction at unit concentration of the reactant^30^. The differential equation in (2) can be integrated using the initial condition [*A*]_0_ at *x* = 0, obtaining the well-known kinetic reaction model,

3 $$\int\limits_{{\left[ A \right]_{0} }}^{\left[ A \right]} {\frac{d\left[ A \right]}{{\left[ A \right]}} = } - \int\limits_{0}^{t} {Kdt} \quad \Rightarrow \quad \left[ A \right] = \left[ A \right]_{0} e^{ - Kt}$$

The differential equation in (2) can be extended to any integer positive order *n* as follows,

4 $$r = \frac{d\left[ A \right]}{{dt}} = - K\left[ A \right]^{n}$$

Also, the decay rate of a reactant in Eq. (4) can be integrated using the initial condition [*A*]_0_ at *x* = 0, obtaining the *n*-th kinetic reaction model,

5 $$\int\limits_{{\left[ A \right]_{0} }}^{\left[ A \right]} {\frac{d\left[ A \right]}{{\left[ A \right]^{n} }} = } - \int\limits_{0}^{t} {Kdt} \quad \Rightarrow \quad \left[ A \right] = \left[ {\left( {n - 1} \right)Kt + \left[ A \right]_{0}^{1 - n} } \right]^{{\frac{1}{1 - n}}} \quad \overbrace { \Rightarrow }^{n = 2}\quad \left[ A \right] = \frac{1}{{Kt + \left[ A \right]_{0}^{ - 1} }}$$

Note that considering Eq. (2), the Eq. (4) can be written as:

6 $$r = \frac{d\left[ A \right]}{{dt}} = - \left\{ {K\left[ A \right]^{n - 1} } \right\}\left[ A \right] = K_{1} \left( {\left[ A \right]} \right)\left[ A \right]$$

Therefore, assuming the same initial rate of the reaction for unit concentration, i.e., *K*, the kinetic reaction model of higher order allows considering a decrease of the decay rate with concentration. For example, looking at the Eq. (6), the second order corresponds to a reaction rate characterized by a reaction rate, *K*_*1*_, linearly decreasing with substance concentration. Note that the decreasing of the reaction rate can be also calculated using the reaction and the reactant substances inside a second order scheme. However, the use of a second order kinetic model with the reaction substance only does not impair the generality of the work with respect to the purposes previously reported^31^.

### Chlorine decay modelling

As previously reported, the chlorine transport and decay were assumed considering the first or second order kinetic reaction without impairing the generality of the methodology and results. Therefore, the right side of Eqs. (3) and (5), having *n* = 1 and *n* = 2 respectively, were used, where *C*_*k*_ = [A] corresponds to the chlorine concentration along the *k*-*th* pipe of the network.

The calculation started from the velocity field using a novel Lagrangian scheme to model the chlorine transport and decay in the network. One relevant novelty of the water quality modelling relates to the unlimited number of parcels allowed in each pipe depending on the maximum travel time, only restricted by the memory and storage capacity of the calculation environment. Another novelty is the mapping of the parcels changing pipes velocity over time, while using a robust approach that keeps the spatial mass distribution of the substance by interpolating the concentrations when the masses are aggregated or split. Overall, the method here used allows an accurate calculation of the chlorine concentration in each node. Further details can be found in^1^. The reaction rate parameter *K* is the straightforward sum of the bulk decay and the wall decay coefficients, which disregards the effect of the variation of the wall decay coefficient for different fields of velocity, as well as the surface area available for reaction and the mass-transfer between the bulk fluid and the wall^10^. We assumed during numerical experiments to be used by EPR three values *K* = {2,2.5,3} day^-1^.

### Evolutionary polynomial regression

As previously reported, EPR is a *genetic programming*^4^ strategy of machine learning allowing to get from data model formulas, i.e., symbolic/explicit models of data, through genetic algorithm (EPR-MOGA). The general structure of EPR is^2,3^:

7 $$Y = \sum\limits_{j = 1}^{m} {f\left( {{\mathbf{X}},a_{j} } \right)} + a_{o}$$

where *Y* is the estimated value of the target; *m* is the number of terms of the expression (bias excluded); *f* is a function constructed by the process; *a*_*j*_ is an adjustable parameter for the *j-th* term; **X** is the matrix of input variables, each raised to an exponent, and *a*_*o*_ is an optional bias.

EPR-MOGA follows a two-step procedure: (a) structure identification and (b) parameter estimation^2^. The first step consists in finding the optimal combination of vectors of independent variables and related exponents simultaneously. The optimization is undertaken using a multi-objective genetic algorithm, which includes three objectives: maximization of the fitness function, as well as minimization of the number of polynomial coefficients and the number of inputs^2,3^. Moreover, since the candidate exponents are defined by the user, this allows for dismissing some of the inputs when zero is included, thus only the significant variables for each output are added to the model. The second step applies the linear least squares method to find the values of the parameters of each term based on the minimization of the sum of squared errors^2^.

Since EPR can select independent variables (model input) which are treated as hypothesis, the user can build other inputs, by aggregating the original ones, to introduce prior physical knowledge as in this case. As well as the inputs, the selected function, exponents, and the maximum number of terms of the EPR model are hypothesis, i.e. candidates to modelling result. The EPR strategy, then, generates understandable and less complex models in term of parameters in contrast with artificial neural networks. The pressure to minimize the model complexity in the MOGA strategy allows avoiding overfitting, being EPR a balance between regressive capability and search for simple models.

After the EPR run, the user can select a model equation among the Pareto optimal based on the physical insight about the modelling phenomenon and the added value of the model complexity with respect to the fitting to data. In this sense, EPR supports knowledge discovery with a data-based approach as in the present effort. In the modelling classification framework, EPR can be classified as a grey-box method because the model is built based on physical insight, and it presents the relationship between inputs and output with an explicit expression, whose consistency can be easily analysed and understood^32^. Hence, this is a comparative advantage of EPR in contrast with black-box techniques such as artificial neural networks.

Regarding the present study, preliminary tests performed with several structures revealed that the best generalization performance was achieved using the following structure, among those available in EPR:

8 $$Y = a_{0} + \sum\limits_{j = 1}^{m} {a_{j} \cdot \left( {{\mathbf{X}}_{1} } \right)^{{{\mathbf{ES}}(j,1)}} \cdot ... \cdot \left( {{\mathbf{X}}_{k} } \right)^{{{\mathbf{ES}}(j,k)}} }$$

where *k* is the number of inputs and **ES** is a matrix of exponents.

## Application of the proposed approach to test WDNs

**Figure 1:**
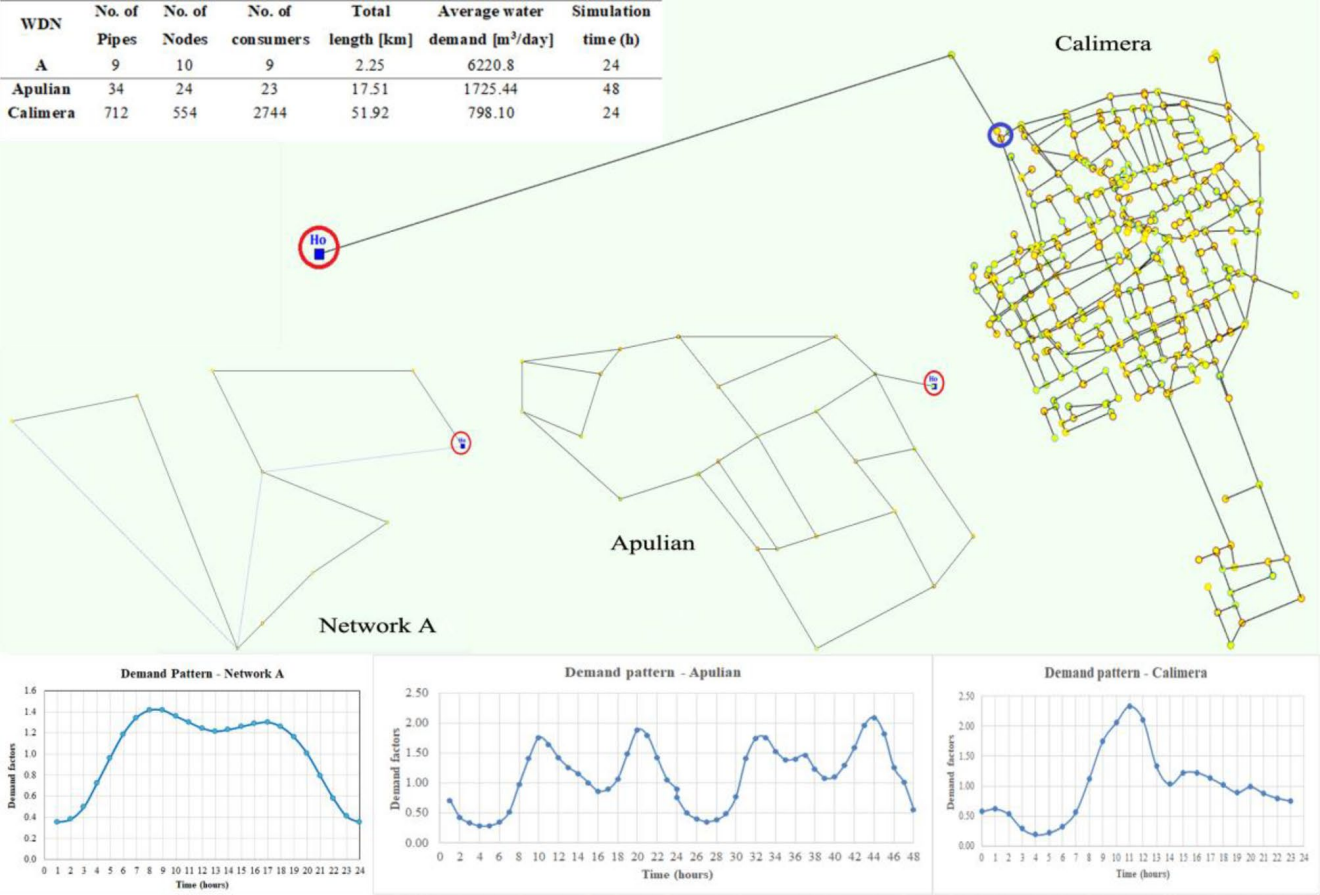
Network A, Apulian WDN, Calimera WDN: topology, demand patterns and attributes.

The attributes of the WDNs used for this research are summarized in Table 1, where the case studies are ordered from smallest to largest and most complex. The hydraulic models of the networks and the corresponding demand patterns are shown in Fig. 1 for Network A, Apulian WDN and Calimera WDN. The reservoir is indicated with a red circle.

**Table 1:** EPR parameters of the two tests: A and B for first and second order kinetics.

Parameter	Value
Inputs	${\mathbf{A}}_{1st - order} = \left[ {Q_{i} ,v_{i} ,C_{i} ,K,Age_{j} ,C_{i} \cdot \exp \left( { - K \cdot Age_{j} } \right)} \right]$ ${\mathbf{A}}_{2nd - order} = \left[ {Q_{i} ,v_{i} ,C_{i} ,K,Age_{j} ,C_{i} \cdot \exp \left( { - K \cdot Age_{j} } \right),\left( {K \cdot Age_{j} + C_{i}^{ - 1} } \right)^{ - 1} } \right]$
	${\mathbf{B}}_{1st - order} = \left[ {Q_{i} ,v_{i} ,C_{i} ,K,SP_{j} ,C_{i} \cdot \exp \left( { - K \cdot SP_{j} } \right)} \right]$ ${\mathbf{B}}_{2nd - order} = \left[ {Q_{i} ,v_{i} ,C_{i} ,K,SP_{j} ,C_{i} \cdot \exp \left( { - K \cdot SP_{j} } \right),\left( {K \cdot SP_{j} + C_{i}^{ - 1} } \right)^{ - 1} } \right]$
Outputs	$C_{j}$
Bias	No
Maximum # of candidate monomials	3
Candidate exponents	[-1, -0.5, 0, 0.5, 1]

Note that Network A is a branched system, Apulian WDN is a small size looped network and Calimera WDN is a real network containing both branches and loops. As shown in Fig. 1, the operative cycle for Network A and Calimera WDN is 1 day, while it is 2 days for Apulian WDN.

As reported in the introduction section, the assumption about consumers’ demand stationarity is determine the timestep of hydraulic modelling. For the aim of the analysis, the timestep equal to one hour is a good accuracy; Therefore, the hydraulic analysis refers to a consumers’ demand varying hour by hour, according to the demand factors in the demand patterns of Fig. 1.

For all the networks, the dataset for EPR-MOGA was generated using the kinetic model of first order, while the kinetic model of second order was used for Calimera WDN only, because it was useful to verify the identifiability of the kinetic model. The assumption of a reaction rate parameter, constant for all the pipes, was instrumental to build formula models having a single parameter as input for each calculation. The verification of the EPR-MOGA models using a more realistic and variable reaction rate parameter and the related discussion was successively performed. Therefore, the dataset for EPR-MOGA was generated combining three realistic values of *K* = {2, 2.5, 3} day^-1^ with three chlorine doses, *C*_*i*_ = {0.3, 0.4, 0.5} mg/L at the reservoir for the first two networks, and *C*_*i*_ = {0.5, 0.75, 1.0} mg/L at the entrance of the water system for Calimera WDN. Note that higher doses were chosen for Calimera WDN to be able to see the decay better, being a greater size hydraulic system. Thus, nine water quality simulations were executed to create the dataset for EPR-MOGA, and the analysis was performed using the timestep of the water quality analysis equal to 1 min.

As reported, a selected formula model provided by EPR-MOGA was tested and discussed using Calimera WDN, performing a new water quality analysis using more realistic values of the reaction rate parameter *K,* depending on the roughness attributes of each pipe, while the chlorine dose was set equal to 0.5 mg/L. To the purpose of setting a more realistic reaction rate parameter, the method indicated by Rossman in EPANET tutorial is used^31^. The reaction rate parameter is given by the summation of the bulk decay parameter, which was set equal to 0.15 day^-1^ as reported in^15,33^ and the wall decay parameter, which depends on a parameter *F* and the pipe roughness^31^. In this work, the parameter F, named *wall reaction—pipe roughness coefficient*, is set equal to {4, 5, 6}.

**Figure 2:**
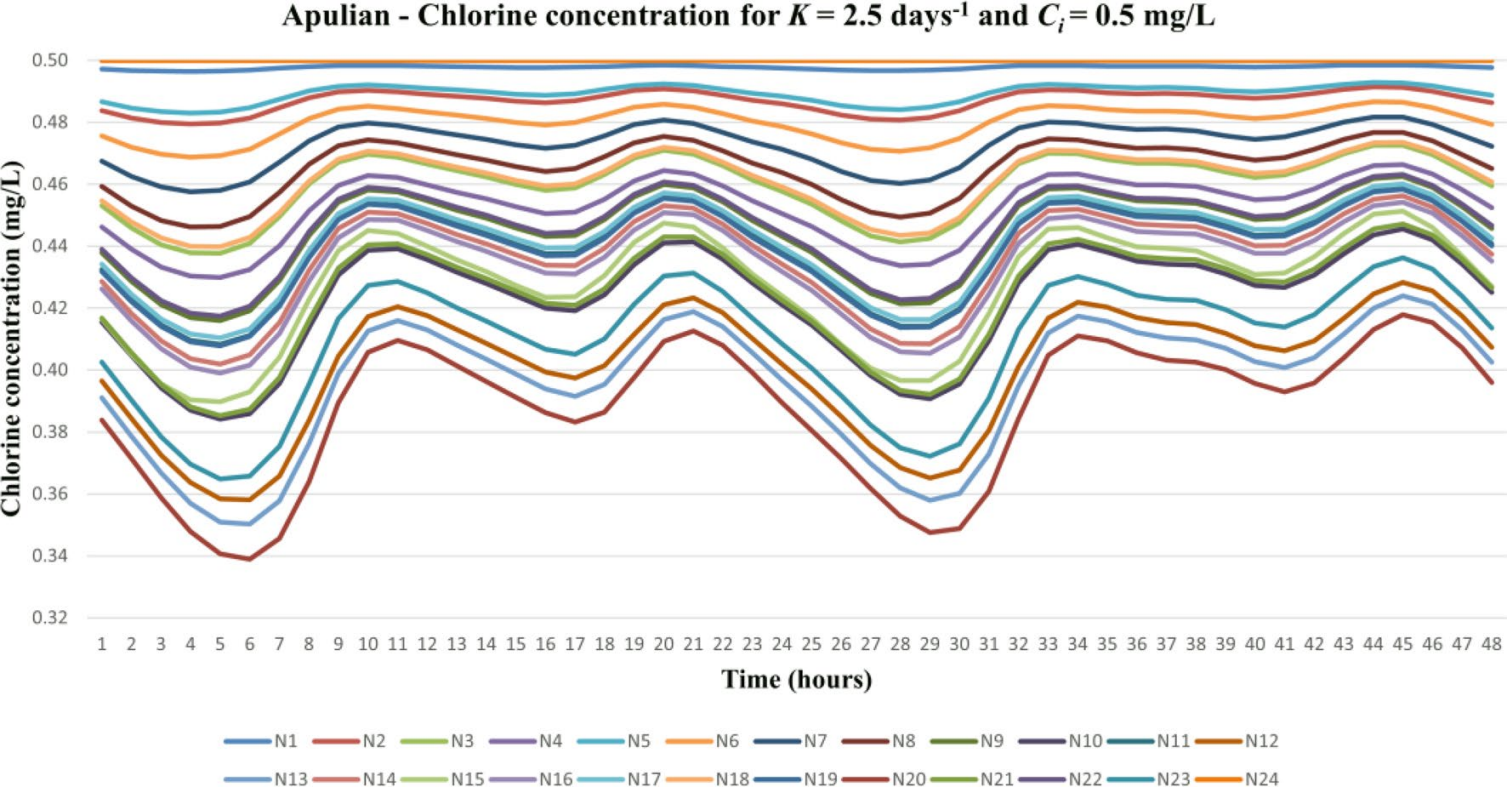
Apulian network—Chlorine concentration for *K* = 2.5 day^-1^ and *C*_*i*_ = 0.5 mg/L.

For illustrative purposes, the results with an overall decay coefficient equal to *K* = 2.5 day^-1^, and a chlorine dose of *C*_*i*_ = 0.5 mg/L for the Apulian WDN are shown in Fig. 2, where N1, N2, …, etc. indicate the number of nodes in the network. The plot demonstrates a regular and stable tendency without spikes, which proves the accuracy of the algorithm.

The relevant hydraulic and water quality variables of the nine water quality simulations with a unique hydraulic basis (pipes velocity field) were assembled as input (independent variables) of the EPR-MOGA dataset, while the computed nodal chlorine concentration over time as output (target). However, the variable nodal water age, i.e., the time the water travels from the source node to each node of the network, was independently computed for a unique velocity field. Since the calculation of water age is computationally intensive, the shortest paths based on the pipes velocity field from the source node to each node of the networks were also computed with the aim of exploring the possibility to surrogate the independent variable water age^28^.

Table 1 reports EPR setting; the two set of inputs, namely **A** and **B**, refers to the use of nodal water age, *Age*_*j*_, or nodal shortest paths, *SP*_*j*_, while a second set for **A** and **B** is useful for Calimera WDN to assess the kinetic model order.

The independent variables (inputs) used for EPR are shown in Table 1 for both first and second order kinetics, where *Q*_*i*_ [m^3^/s] and *v*_*i*_ [m/s] are the flow rate and velocity in the outcoming pipe from the reservoir, *C*_*i*_ is the chlorine dose at the reservoir [mg/L]; *K* is the reaction rate parameter [day^−1^] for first order and second order kinetics, respectively; *Age*_*j*_ is the water age at the j*-*th node [h], *SP*_*j*_ is the travel time in the shortest path(s) for the *j-th* node [h] and *C*_*j*_ is the chlorine concentration at the j-*th* node. The units of the reaction rate parameter were changed to *h*^*-1*^ when used for consistency of the other units.

About the selection of the number of terms for EPR-MOGA models, it is here selected a maximum number of terms *m* = 3, since a greater number does not significantly influence the result because EPR-MOGA searches for parsimonious models, i.e., has an internal pressure to select models with few monomials because of the optimization strategy (see paragraph 2.4). The bias was set null because an offset in the decay process was not assumed.

The candidate exponents are instrumental to the aim of data modelling. In this case, see Table 1, we decided to search for linearity and square root models (direct or inverse) after considering some test runs, which indicated the selected exponents as the most relevant for the specific process at stake. Without impairing the EPR-MOGA effectiveness in exploring the models’ space, the selection of candidate exponents in Table 1 refers to the last run that avoid obtaining an excessive number of Paretian models.

The EPR-MOGA findings about the decay mechanism in the pipes network domain were discussed for the three networks. The results from the simple branched network to the most complex (Calimera WDN) passing through a simple looped one, allows explaining the reasoning of the finding related to the relevance of the hydraulic velocity shortest paths. In fact, the substance decay between a source node and any other node in the network is mostly dependent on the decay process in the shortest paths which transfer the major amount of water between nodes.

Then, for Calimera WDN test data, i.e., unseen for EPR model construction, were used to discuss the influence of the variability of *K* for each pipe in real networks.

## Modelling results and discussion on mechanism of transport and decay

As explained above, running EPR-MOGA returns a set of Pareto models (i.e., all non-dominant to each other) having different complexity and accuracy on the training inputs. In what follows, only the models considered most significant also from the physical consistency point of view are shown in the tables and discussed. The whole set of Pareto models for each case study is available in the Supplementary file. The Mean Absolute Error (MAE) of selected expressions for each WDN was plotted to analyse the spatial distribution of the accuracy of the EPR-MOGA models depending on the inputs, i.e., water age (**A**), or travel time in the shortest path(s) (**B**).

### Network A

Table 2 shows the selected models obtained with first order data for Network A, that is the simplest WDN of the cases of study. In this sense, it is a single-branch network for which the water age and chlorine decay calculations are trivial.

**Table 2:** Network A—selected EPR models with first order data.

Test inputs	Expression	$R^{2} \left( \% \right)$
A_1st-order_	$C_{j} = C_{i} \cdot \exp \left( { - K \cdot Age_{j} } \right)\quad (9)$	$100$
B_1st-order_	$C_{j} = 1.0002 \cdot C_{i} \cdot \exp \left( { - K \cdot SP_{j} } \right)\quad (10)$ $C_{j} = 0.9992 \cdot C_{i} \cdot \exp \left( { - K \cdot SP_{j} } \right) + 0.001228 \cdot SP_{j}$	$99.933$ $99.936$

The extreme simplicity of this network can help in understanding how much a symbolic machine learning technique, EPR-MOGA in our case, can "catch" the phenomenon by data. This is the case of models in Table 2, both from the point of view of accuracy (*R*^*2*^ almost equal to 1), and from that of physical consistency, i.e., comparing Eq. (9) with the relevant physical-based model, i.e., the first order kinetic reaction model, see Eq. (3).

The similarity and accuracy of Eqs. (9) and (10) confirms the modelling choice of considering water age and travel time in the shortest path(s) as equivalent for the purposes of the present work. Moreover, the results confirm the relevance of the exponential terms exp(*-K∙Age*_*j*_) and exp(*-K∙SP*_*j*_) as aggregate inputs to introduce prior physical knowledge about first-order chlorine decay. The hydraulic variables *Q*_*i*_ and *v*_*i*_ are not contained in neither of the expressions, likely because their influence is already considered in the estimation of travel time.

**Figure 3:**
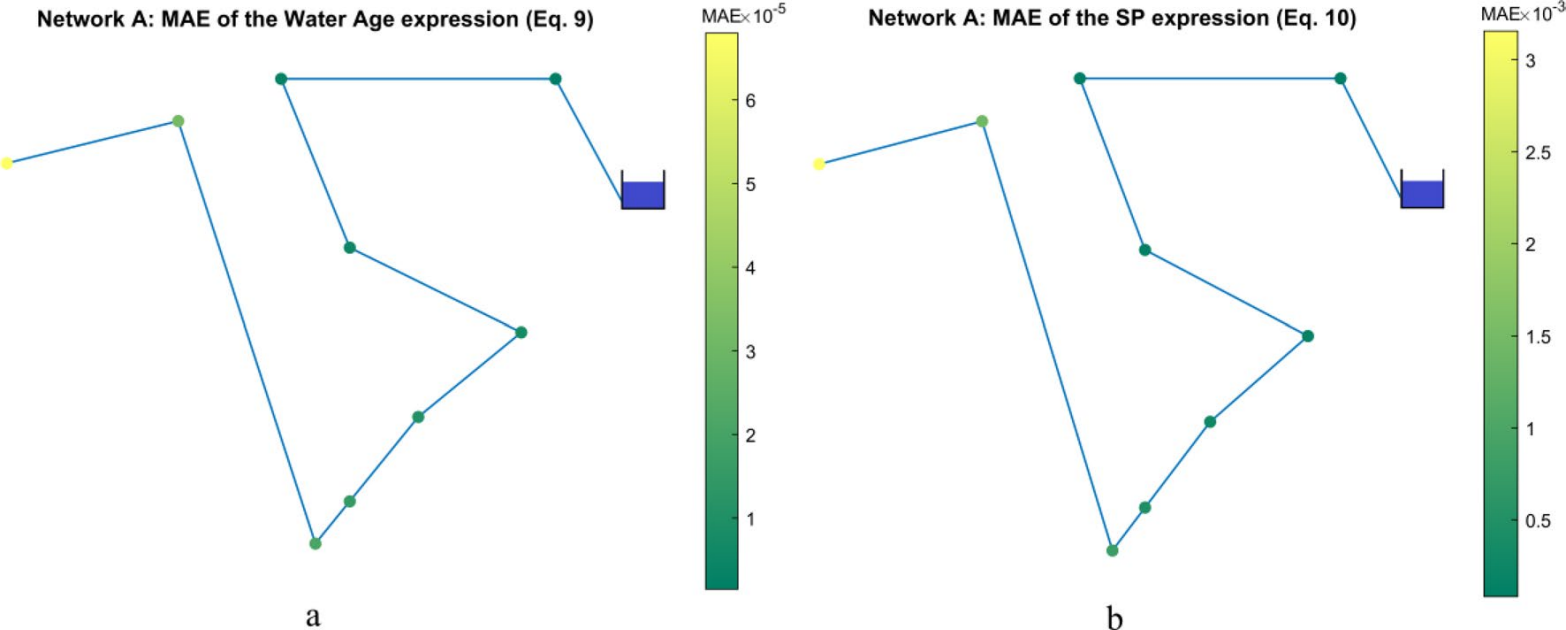
**(a**) Network A: MAE of Eq. (9). (**b**) Network A: MAE of Eq. (10).

The distribution of the MAE for the models in Table 2 is shown in Fig. 3. The MAE values are low for all the nodes, which indicates a good performance along the network.

### Apulian WDN

Table 3 contains the EPR results for the Apulian WDN. It is a simple network with loops and only one branch corresponding to the pipe feeding the system from the single reservoir. The aim of this case study is to start understanding the role of the loops, although in a small network. Like in the case of Network A, the coefficients of determination of all the expressions are high, which indicates a satisfactory performance of all Paretian models despite the loops.

**Table 3:** Apulian WDN—selected EPR models with first order data.

Test inputs	Expression	$R^{2} \left( \% \right)$
A_1st-order_	$C_{j} = 1.0015 \cdot C_{i} \cdot \exp \left( { - K \cdot Age_{j} } \right)\quad (11)$	99.957
	$C_{j} = 0.97946 \cdot C_{i} \cdot \exp \left( { - K \cdot Age_{j} } \right) + 0.019636 \cdot C_{i} \quad (12)$	99.963
B_1st-order_	$C_{j} = 0.992 \cdot C_{i} \cdot \exp \left( { - K \cdot SP_{j} } \right)\quad (13)$	99.359
	$C_{j} = 1.042C_{i} \cdot \exp \left( { - K \cdot SP_{j} } \right) - 0.0443 \cdot C_{i} \quad (14)$	99.382

Also in this case, the exponential terms alone are enough to obtain highly performing and physically consistent models, both for inputs **A**_***1st-order***_ and **B**_***1st-order***_. In fact, models that have an additional term to the exponential one, see Eqs. (12) and (14), do not gain significantly in accuracy.

Moreover, although the Apulian WDN incorporates secondary paths, between the source node and the others, the single exponential model, Eq. (13), is still valid when the travel time in the shortest path(s) is used. Hence, the travel time of the shortest path(s) prevails over the secondary paths.

**Figure 4:**
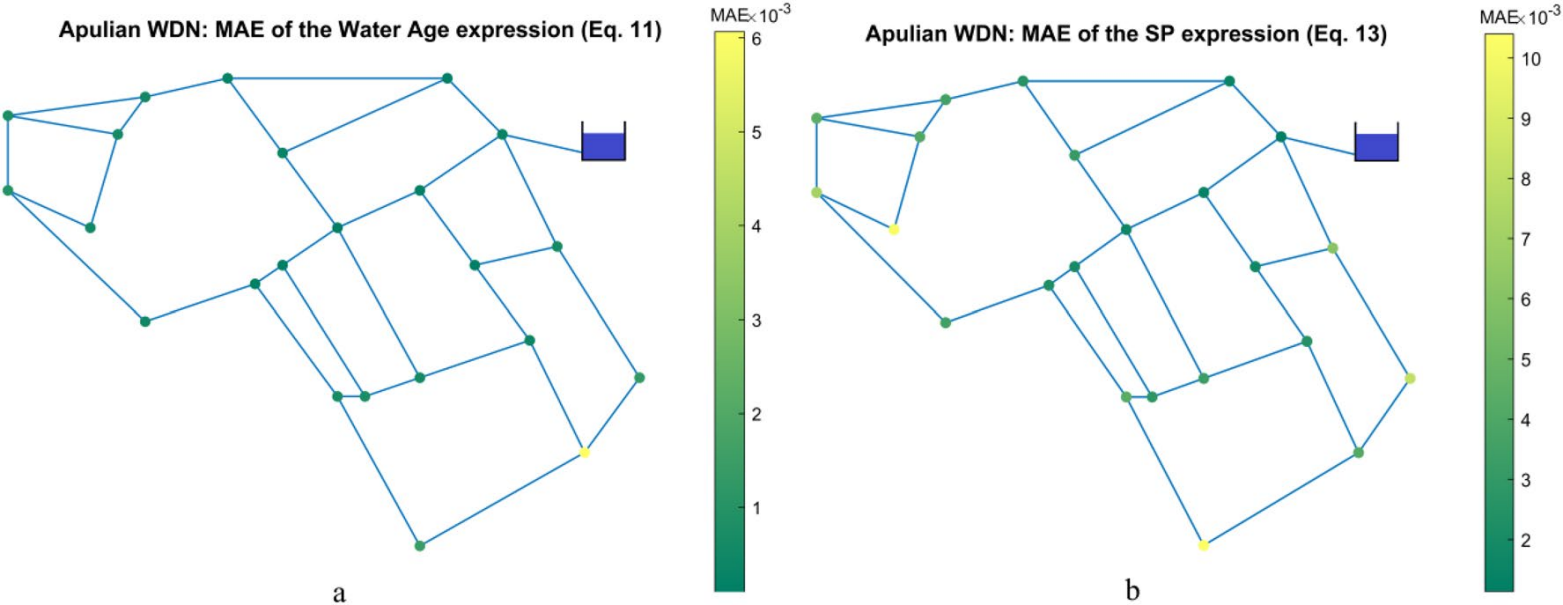
**(a**) Apulian WDN: MAE of Eq. (11). (**b**) Apulian WDN: MAE of Eq. (13).

The distribution of the MAE for the expressions with test inputs **A**_*1st-order*_ and **B**_*1st-order*_ in the Apulian WDN is shown in Fig. 4. The MAE values are generally low and there are no significant outliers throughout the network.

### Calimera WDN

The Calimera WDN was used with the aim of demonstrating the proposed approach on a real water system composed by loops and branches, higher water ages and more secondary paths, between the source node and the others, with respect to the shortest paths.

The selected EPR-MOGA models with first and second order kinetics are shown in Table 4. The candidate inputs for second order data are shown in Table 1.

**Table 4:** Calimera WDN – Selected EPR models with first order and second order kinetics data.

Test inputs	Expression	$R^{2} \left( \% \right)$
A_1st-order_	$C_{j} = 1.009 \cdot C_{i} \cdot \exp \left( { - K \cdot Age_{j} } \right)\quad \quad (15)$	99.763
	$$\begin{gathered} C_{j} = 0.0272 \cdot \left( {C_{i} \cdot \exp \left( { - K \cdot Age_{j} } \right)} \right)^{0.5} \hfill \\ + 0.972 \cdot C_{i} \cdot \exp \left( { - K \cdot Age_{j} } \right)\quad (16) \hfill \\ \end{gathered}$$	99.783
B_1st-order_	$C_{j} = 0.90275 \cdot C_{i} \cdot \exp \left( { - K \cdot SP_{j} } \right)\quad (17)$	91.025
	$C_{j} = C_{i} \cdot \exp \left( { - K \cdot SP_{j} } \right)\left( {1.138 - 0.258 \cdot SP_{j}^{0.5} + 0.0606 \cdot SP_{j} } \right)\quad (18)$	91.713
A_2nd-order_	$C_{j} = 1.0068 \cdot \left( {K \cdot Age_{j} + C_{i}^{ - 1} } \right)^{ - 1} \quad (19)$	99.758
	$C_{j} = 0.99678 \cdot \left( {K \cdot Age_{j} + C_{i}^{ - 1} } \right)^{ - 1} + 0.0067055 \cdot C_{i} \quad (20)$	99.767
B_2nd-order_	$C_{j} = 0.94307 \cdot \left( {K \cdot SP_{j} + C_{i}^{ - 1} } \right)^{ - 1} \quad (21)$	93.938
	$C_{j} = 1.0326 \cdot \left( {K \cdot SP_{j} + C_{i}^{ - 1} } \right)^{ - 1} - 0.10412 \cdot C_{i} \cdot \exp \left( { - K \cdot SP_{j} } \right)\quad (22)$	94.112

The first evidence observing the models in Table 4 is that, as for Network A and Apulian WDN, also in this case have been generated models that have a notable physical consistency, i.e., comparing Eqs. (15) and (17) with the relevant physical-based model, i.e., the first order kinetic reaction model, see Eq. (3). For second order kinetics have been also produced models that can be reasonably superimposed on their physically based counterparts, i.e., comparing Eqs. (19) and (21) with the second order kinetic reaction model, see Eq. (5). This means that the proposed approach based on EPR-MOGA can distinguish between first and second order decay process since the selected inputs within the monomials correspond to the relative analytical solution, e.g. first or second order equations. As an exception, Table 4 reports Eq. (22), that incorporates both type of terms with little difference in the R^2^ in contrast with Eq. (21). Therefore, EPR may be useful to identify the type of decay that best fits measured data.

Even though the Calimera WDN is larger and more complex than Network A and the Apulian WDN, those expressions containing a single term keep have a satisfactory performance. This indicates that the relevant inputs and the structure of the formulas to describe the first or second order (chlorine) decay are not substantially influenced by the size and topology of the network. Additionally, for all the input set analysed, the difference between the R^2^ of the simplest formulas and the expressions with a greater number of terms is below 1%. Therefore, the decay mechanism throughout a WDN can be reasonably modelled with simple EPR-MOGA models with a satisfactory degree of accuracy even for increasingly complex WDNs.

Note that the R^2^ values of the formulas that include water age are slightly higher in contrast to those of the expressions that contain the travel time in the shortest path(s). Hence, water age seems to be a more accurate descriptor of the first-order chlorine decay phenomenon for more complex WDNs. There are two main reasons that could explain this fact: (a) the dominant shortest path changes over time; and (b) the number of secondary routes with a significant contribution to the concentration of each node is larger when the size and complexity of the network increase, whereas a single dominant shortest path is found for smaller networks.

It should also be noted that the expressions that include the travel time in the shortest path(s) for second order kinetics, i.e., Eqs. (21) and (22), have a slightly higher R^2^ than those corresponding to the first order kinetics, i.e., Eqs. (17) and (18). This means that the decrease in the prediction accuracy when using the travel time in the shortest path(s) instead of water age for first order kinetics is greater than that for second order equations. Hence, this could mean that the travel time in the shortest path(s) is a better surrogate for water age when applying second order kinetics.

**Figure 5:**
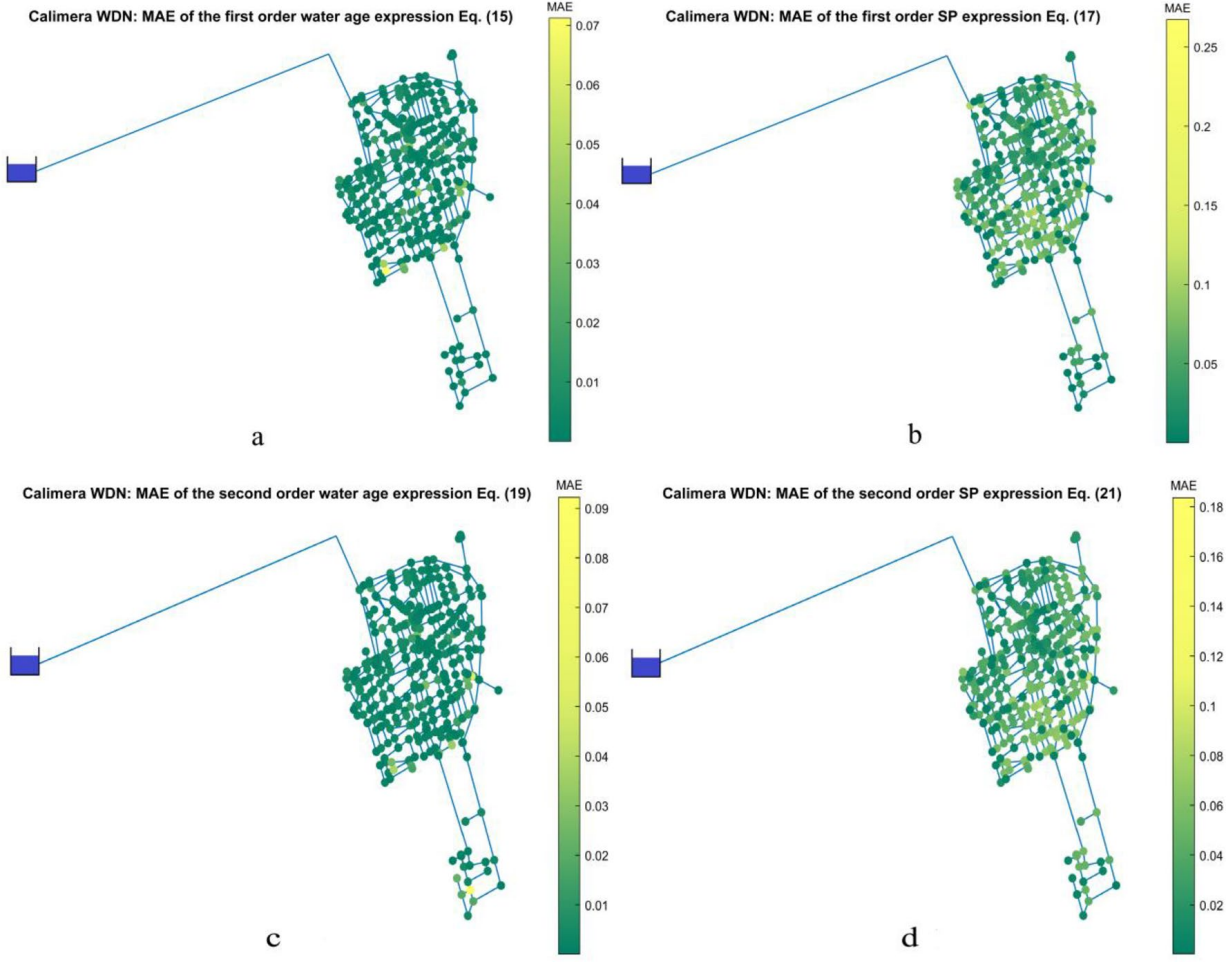
**(a**) Calimera WDN: MAE of Eq. (15); (**b**) MAE of Eq. (17); (**c**) MAE of Eq. (19); (**d**) MAE of Eq. (21).

The distribution of the MAE for the expressions with test inputs A and B for first and second order decay in the Calimera WDN is shown in Fig. 5. Like the other case studies, the MAE values are low and there are no representative outliers throughout the network. Figures 5a,c show very few nodes in which Eqs. (15) and (19) have an unsatisfactory performance:the nodes with the greatest errors for second order kinetics using water age are in the terminal branches of the network, Figs. 5c, while for first order equations they are in the peripheral loops, Figs. 5a. Conversely, the distribution of MAE for the expressions that use the travel time in the shortest path(s) is similar for both first and second order kinetics, as displayed in Fig. 5b,d.

## Testing findings and results

The chlorine reaction rate parameters used for testing the EPR-MOGA formula models on the test set (made of unseen data) was defined with four different alternatives:

- *K*_*net*_, which is the weighted average of the overall decay coefficients of the pipes of the network, except for the ones located upstream of the chlorine input node, with the length as the weighting factor.
- *K*_*SPvar*_, that is the weighted overall decay coefficient of the pipes in the shortest path from the chlorine input node to each node for every timestep of the analysis. This coefficient changes over time since the shortest path varies according to the field of velocities in the network.
- *K*_*mSPn*_, which is the mean *K*_*Spvar*_ for each node over time.
- *K*_*mSP*_, that is the mean of the *K*_*SPvar*_ matrix excluding the chlorine input node and the upstream nodes.

Note that these alternatives are equivalent when reaction rate is constant *K*. For this reason, EPR used to generate symbolic models with the constant *K*, while the discussion on the meaning in the formula is studied before using unseen data and water quality analysis with variable *K*. Table 5 shows the MAE for each alternative of the reaction rate parameter applied in Eqs. (15) and (19) for the Calimera WDN with first and second order data, respectively. Without impairing the generality of the results, the choice of Eqs. (15) and (19) depends on they have a slightly higher accuracy than those using the travel time along the shortest path(s).

**Table 5:** MAE using each reaction rate parameter in Eq.s (15) and (19) for Calimera WDN.

K	MAE—First order			MAE—Second order		
	*F* = 4	*F* = 5	*F* = 6	*F* = 4	*F* = 5	*F* = 6
K_net_	0.00671	0.00746	**0.00806**	0.00478	0.00517	**0.00555**
K_SPvar_	0.00514	0.00506	0.00496	0.00554	0.00545	0.00538
K_mSPn_	0.00509	0.00499	**0.00489**	0.00550	0.00540	0.00533
K_mSP_	0.00582	0.00569	0.00552	0.00592	0.00583	**0.00576**

Significant are in value [bold].

For first order data, the best performance was obtained using *K*_*mSPn*_, followed by *K*_*SPvar*,_ *K*_*mSP*_ and *K*_*net*_. The MAE of *K*_*mSPn*_ is slightly lower as the *F* parameter increases, which is due to a steeper decay that causes lower chlorine concentrations and absolute errors. Moreover, the MAE of *K*_*SPvar*_ is greater than the MAE of *K*_*mSPn*_, although the former describes the variation of the overall decay coefficient over time in detail, and thus it would be expected to have a lower error. This fact is due to the time lag between the computation of the shortest path depending on the field of velocity at a given instant and the arrival of such water parcels into each node. Hence, *K*_*mSPn*_ has a better performance since it encapsulates the changes of the shortest paths over time.

For second order data, the best performance was obtained using *K*_*net*_, followed by *K*_*mSPn*_, *K*_*SPvar*_ and *K*_*mSP*_. *K*_*mSP*_ is lower than *K*_*net*_, thus the chlorine concentrations computed with the former are greater than those calculated with the latter. Therefore, *K*_*mSP*_ tends to overestimate chlorine concentrations, which indicates that the influence of the decay coefficients in secondary paths should also be considered for a better estimation. Despite this, the difference in MAE between the results with both constants is not significant and it decreases as *F* increases.

**Figure 6:**
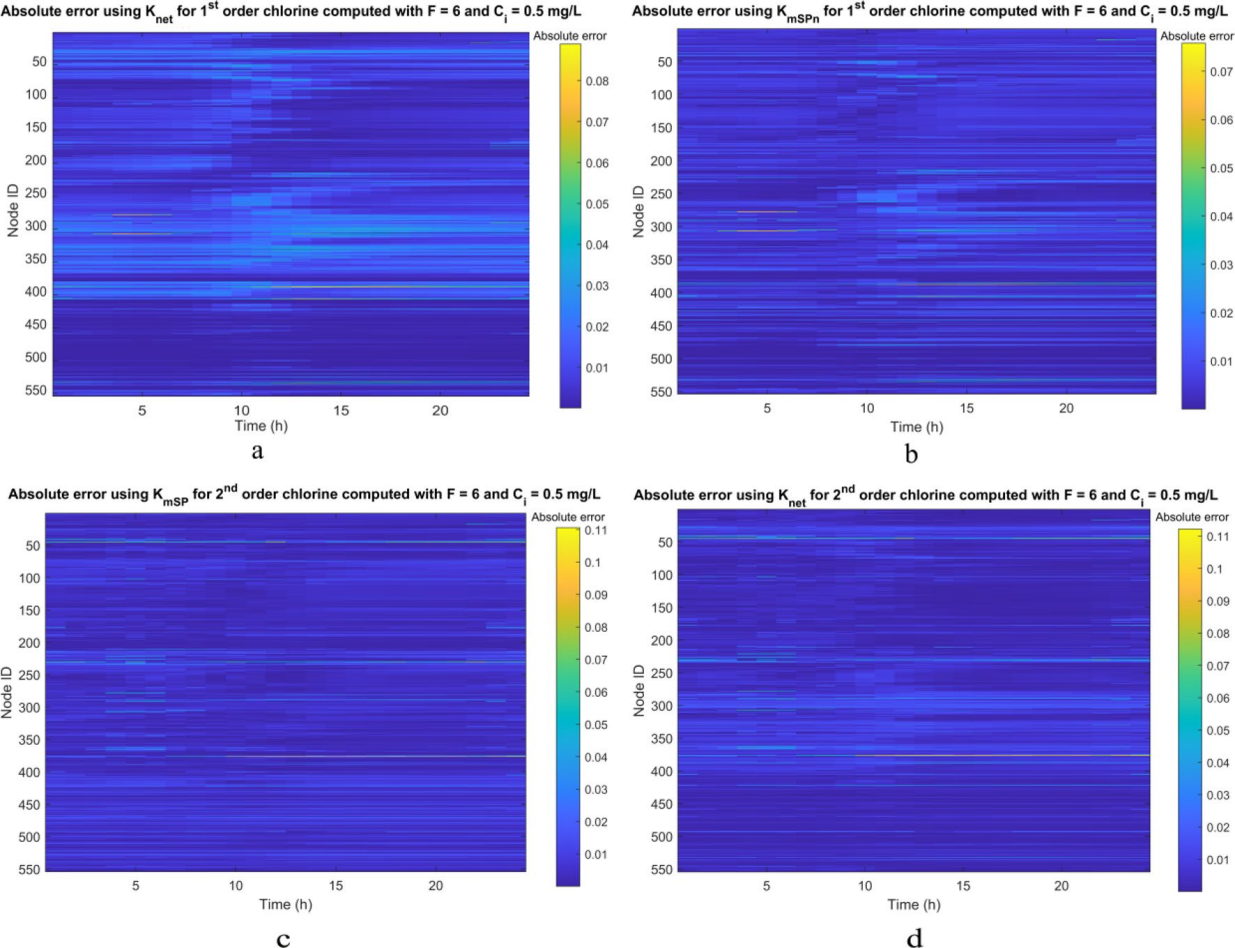
**(a**) Calimera WDN: AE with K_net_ for first order chlorine with *F* = 6. (**b**) AE with K_mSPn_ for first order chlorine with *F* = 6; (**c**) AE with K_net_ for second order chlorine with *F* = 6; (**d**) AE with K_var_ for second order chlorine with *F* = 6.

Figure 6a,b show the temporal and nodal variation of the Absolute Error (AE) in the Calimera WDN using *K*_*net*_ and *K*_*mSPn*_ in Eq. (15), respectively. Figure 6a shows that the performance of *K*_*net*_ has a predominant spatial pattern since low errors during all the simulation time are concentrated in the group of nodes with IDs around 400 and 550, whereas other nodes have low errrors only for a part of the simulation time, and medium errors are also frequent. In contrast, Fig. 6b displays lower errors more uniformly distributed in time and space, which indicates a greater generalization capacity.

Similarly, Figs. 6c,d display the temporal and spatial variation of the AE in the Calimera WDN using *K*_*mSP*_ and *K*_*net*_ in Eq. (19), respectively. In this case, altough they have respectively the worst and best performance in terms of MAE, thet can be considered substantially comparable, as clear from the Figs. 6c,d. Figure 6c shows slightly higher errors for nodes with IDs between 400 and 550, which are generally located in terminal branches of the network. Conversely, Fig. 6d9 only exhibits a slightly poor performance for nodes with IDs between 300 and 400, that are located in loops with significant travel times.

## Conclusions

The aim of the presented research was to analyse the intrinsic mechanism of the substance (i.e., chlorine) transport in WDNs, i.e. their pipes network domain, using the symbolic machine learning, named Evolutionary Polynomial Regression, EPR-MOGA, which provides “synthetic” formulas for models from data. This study showed that similar EPR formulas are found for WDNs with distinctive hydraulic, geometrical, and topological attributes. The accuracy of the proposed approach is not impaired as the size of the network grows, since the fitness of the best formula for each WDN is similarly satisfactory among the case studies. Moreover, it was proved that the EPR formulas may be generalized to WDNs with variable reaction rate parameters, being possible to model the behaviour of first and second order chlorine decay kinetics throughout a WDN by a single and simple polynomial expression with a suitable degree of accuracy.

The potential applications of such a formula are mainly related to modelling, calibration (such as the assessment of the reaction rate parameters), and optimization purposes. Furthermore, symbolic machine learning like EPR may be used to identify the type of kinetics that best fits measured data, even for species different from chlorine or multispecies models.

Moreover, this study showed that the “synthetic” formulas for models for first and second order kinetics found with water age inputs had similar performance in comparison with the models that included the travel time in the shortest path(s). In this sense, any of both inputs may be used as a variable to explain chlorine decay in the case of small networks, such as Network A or Apulian WDN, without a significant loss of accuracy, since there is a single dominant shortest path for each node. Regarding large-scale networks such as Calimera WDN, the travel time in the shortest path(s) provides a good approximation, while water age has a slightly better description capacity, likely due to the emergence of relevant secondary paths. Therefore, considering that the calculation of nodal water age is computationally intensive, the results here provided confirm that the alternative approach of surrogating the water age by means of shortest paths based on the velocity field can lead to very good outcomes, both in terms of accuracy and computational costs, for WDNs of different dimensions. Additionally, travel time in the shortest path(s) has a better prediction performance in second order equations in contrast to first order kinetics.

Further studies can investigate the accuracy of surrogating the water age by the travel time on the shortest path(s) based on the characteristic of the network domain of the hydraulic system such the average nodal degree or the density of loops. Such comparison between both parameters should be done also in WDNs with contrasting characteristics, such as multiple reservoirs with different chlorine doses, in which several secondary paths take place, or booster chlorination points.

In the case of multiple reservoirs, further research should study an input strategy for each node with a water trace analysis that allows to divide the network into two types of zones: zones that are fed by a single reservoir, and zones that are fed by more than one reservoir. Furthermore, including booster doses would require performing topological analyses to compute water quality in the influence zone of each booster point.

Additionally, future efforts may explore the estimation efficiency of the travel time in the shortest path(s) and water age for different decay kinetics, given that we could imagine kinetics of order greater than 2 which would be masked by specific kinetics of multiple reactants (so-called multi-species). Future works could analyse the application of the proposed methodology to the decay dynamics with more than one reactant, therefore using the kinetic reaction model of higher order, see Eq. (6) in the paragraph 2.4, to prepare the input dataset in a similar way to what done in the present work for the first and second order kinetic reaction models, as in the supplementary material provided.

### Supplementary Information

Supplementary Information.

## Data Availability

The datasets generated and analysed during the current study are available in the Polytechnic University of Bari OneDrive repository at Using Symbolic Machine Learning to Assess and Model Substance Transport and Decay in Water Distribution Networks. The software that supported this research was EPR-MOGA, a dynamic library which can be used as add-on in MS-Excel®, and it is available from the corresponding author with free of charge licensing.
