# Supplementary material for: Using symbolic machine learning to assess and model substance transport and decay in water distribution networks
Source: Sci Rep. 2024 Feb 8;14:3194. doi: 10.1038/s41598-024-53746-1 (PMC10850479; doi:10.1038/s41598-024-53746-1)
Supplement: Supplementary file 1 — Supplementary Information. [file 41598_2024_53746_MOESM1_ESM.docx]

*Nature Scientific Reports*

Supporting Information for

Using Symbolic Machine Learning to Assess and Model Substance Transport and Decay in Water Distribution Networks

Daniele Biagio Laucelli^1^; Laura Enríquez^2^; Juan Saldarriaga^3^; Orazio Giustolisi^4^*

^1^Polytechnic University of Bari, via Orabona, 4, Italy.

^2^Polytechnic University of Bari, via Orabona, 4, Italy.

^3^Universidad de los Andes, Carrera 1 Este, 19 – 40, Bogotá, Colombia.

^4^Polytechnic University of Bari, via Orabona, 4, Bari, Italy. orazio.giustolisi@poliba.it

**Contents of this file**

Tables S1 to S7

**Introduction**

The Tables S1 to S5 show the EPR polynomial expressions using candidate inputs A, related to water age, and candidate inputs B, related to the travel time in the shortest path(s), for Network A, Apulian WDN and Calimera WDN for first order data, while the Tables S6 and S7 show it for Calimera WDN using second order data. The EPR formulas using candidate inputs A for Network A are not presented herein since they are all equivalent to the single equation presented in the article (Eq. 9).

| Expression | $R^{2}\left(\%\right)$ |
| --- | --- |
| $C_{j}=1.0002\cdot C_{i}\cdot\exp\left(-K\cdot SP_{j}\right)$ | $99.933$ |
| $C_{j}=0.9992\cdot C_{i}\cdot\exp\left(-K\cdot SP_{j}\right)+0.0012285\cdot SP_{j}$ | $99.936$ |
| $C_{j}=1.0017\cdot C_{i}\cdot\exp\left(-K\cdot SP_{j}\right)-0.003809\cdot SP_{j}^{0.5}+0.004081\cdot SP_{j}$ |  |
| $C_{j}=1.0007\cdot C_{i}-0.043898\cdot K\cdot SP_{j}\cdot C_{i}\cdot\exp\left(-K\cdot SP_{j}\right)$ | $99.940$ |
| $C_{j}=C_{i}\cdot\exp\left(-K\cdot SP_{j}\right)\cdot\left(1.0004-0.0495\cdot SP_{j}\right)+0.0453\cdot C_{i}\cdot SP_{j}$ | $99.940$ |
| $C_{j}=1.0004\cdot C_{i}\cdot\exp\left(-K\cdot SP_{j}\right)-0.05354\cdot C_{i}^{0.5}\cdot SP_{j}+0.0512\frac{C_{i}^{0.5}\cdot SP_{j}}{\exp\left(-0.5\cdot K\cdot SP_{j}\right)}$ | $99.941$ |
| $C_{j}=C_{i}\cdot\exp\left(-K\cdot SP_{j}\right)\cdot\left(-0.07967\frac{SP_{j}}{K^{0.5}}+1.0004\right)+0.0731\frac{C_{i}\cdot SP_{j}}{K^{0.5}}$ | $99.942$ |
| $C_{j}=-0.149\frac{C{}_{i}^{0.5}SP_{j}\cdot\left(C_{i}\exp\left(-K\cdot SP_{j}\right)\right)^{0.5}}{K^{0.5}}+1.0004C_{i}\exp\left(-K\cdot SP_{j}\right)+0.1427\frac{C_{i}SP_{j}}{K^{0.5}}$ | $99.941$ |

Table S1. Network A - EPR results – Test B with first order data

| Expression | $R^{2}\left(\%\right)$ |
| --- | --- |
| $C_{j}=1.0015\cdot C_{i}\cdot\exp\left(-K\cdot Age_{j}\right)$ | $99.957$ |
| $C_{j}=0.97946\cdot C_{i}\cdot\exp\left(-K\cdot Age_{j}\right)+0.019636\cdot C_{i}$ | $99.963$ |
| $C_{j}=1.77E-05\frac{1}{Age_{j}}+0.978\cdot C_{i}\exp\left(-K\cdot Age_{j}\right)+0.0209\cdot C_{i}$ | $99.963$ |
| $C_{j}=1.99E-05\frac{1}{Age_{j}}+0.953C_{i}\exp\left(-K\cdot Age_{j}\right)+0.0454C_{i}^{0.5}\left(C_{i}\exp\left(-K\cdot Age_{j}\right)\right)^{0.5}$ | $99.963$ |
| $C_{j}=C_{i}\exp\left(-K\cdot Age_{j}\right)\cdot\left(\frac{0.000349}{Age_{j}^{0.5}}+0.951\right)+0.0477C_{i}^{0.5}\left(C_{i}\exp\left(-K\cdot Age_{j}\right)\right)^{0.5}$ | $99.963$ |
| $C_{j}=9.12E-04\frac{C_{i}}{K\cdot Age_{j}^{0.5}}+0.950C_{i}\exp\left(-K\cdot Age_{j}\right)+0.0480C_{i}\left(\exp\left(-K\cdot Age_{j}\right)\right)^{0.5}$ | $99.963$ |

Table S2. Apulian WDN - EPR results – Test A with first order data

| Expression | $R^{2}\left(\%\right)$ |
| --- | --- |
| $C_{j}=0.992\cdot C_{i}\cdot\exp\left(-K\cdot SP_{j}\right)$ | $99.359$ |
| $C_{j}=1.042\cdot C_{i}\cdot\exp\left(-K\cdot SP_{j}\right)-0.0443\cdot C_{i}$ | $99.382$ |
| $C_{j}=C_{i}\cdot\exp\left(-K\cdot SP_{j}\right)\cdot\left(1.0037-0.0120\cdot SP_{j}^{0.5}\right)$ | $99.390$ |
| $C_{j}=1.0055\cdot C_{i}-0.0505\cdot K\cdot SP_{j}\cdot C_{i}\cdot\exp\left(-K\cdot SP_{j}\right)$ | $99.424$ |
| $C_{j}=\frac{-9.77E-05}{SP}+1.007\cdot C_{i}-0.0509\cdot K\cdot SP_{j}\cdot C_{i}\cdot\exp\left(-K\cdot SP_{j}\right)$ | $99.422$ |
| $C_{j}=C_{i}\left(1.0005+0.0122\cdot SP_{j}^{0.5}\right)-0.0535\cdot K\cdot SP_{j}\cdot C_{i}\cdot\exp\left(-K\cdot SP_{j}\right)$ | $99.429$ |

Table S3. Apulian WDN - EPR results – Test B with first order data

| Expression | $R^{2}\left(\%\right)$ |
| --- | --- |
| $C_{j}=1.009\cdot C_{i}\cdot\exp\left(-K\cdot Age_{j}\right)$ | $99.763$ |
| $C_{j}=0.0272\cdot\left(C_{i}\cdot\exp\left(-K\cdot Age_{j}\right)\right)^{0.5}+0.972\cdot C_{i}\cdot\exp\left(-K\cdot Age_{j}\right)$ | $99.783$ |
| $C_{j}=0.954\cdot C_{i}\cdot\exp\left(-K\cdot Age_{j}\right)+0.0441\cdot C_{i}\cdot\left(\exp\left(-K\cdot Age_{j}\right)\right)^{0.5}$ | $99.792$ |
| $C_{j}=0.955\cdot C_{i}\cdot\exp\left(-K\cdot Age_{j}\right)+0.0428\cdot C_{i}\cdot\left(\exp\left(-K\cdot Age_{j}\right)\right)^{0.5}+0.000121\cdot K$ | $99.793$ |
| $C_{j}=C_{i}\cdot\exp\left(-K\cdot Age_{j}\right)\left(0.940-0.0012\cdot Age_{j}\right)+0.0594\cdot C_{i}\left(\exp\left(-K\cdot Age_{j}\right)\right)^{0.5}$ | $99.793$ |
| $C_{j}=C_{i}\cdot\exp\left(-K\cdot Age_{j}\right)\left(0.933-0.00111\cdot K^{0.5}Age_{j}\right)+0.0664\cdot C_{i}\left(\exp\left(-K\cdot Age_{j}\right)\right)^{0.5}$ | $99.793$ |

**Table S4.** Calimera WDN - EPR results– Test A with first order data

| Expression | $R^{2}\left(\%\right)$ |
| --- | --- |
| $C_{j}=0.90275\cdot C_{i}\cdot\exp\left(-K\cdot SP_{j}\right)$ | $91.025$ |
| $C_{j}=\frac{0.0175}{K}+0.889\cdot C_{i}\cdot\exp\left(-K\cdot SP_{j}\right)$ | $91.069$ |
| $C_{j}=0.887\cdot C_{i}\cdot\exp\left(-K\cdot SP_{j}\right)-\frac{0.0148}{C_{i}^{0.5}}+\frac{0.06105}{K}$ | $91.089$ |
| $C_{j}=C_{i}\cdot\exp\left(-K\cdot SP_{j}\right)\left(0.933-0.01795\cdot SP_{j}^{0.5}\right)$ | $91.073$ |
| $C_{j}=\frac{0.0374}{K}+C_{i}\cdot\exp\left(-K\cdot SP_{j}\right)\left(0.931-0.0333\cdot SP_{j}^{0.5}\right)$ | $91.190$ |
| $C_{j}=C_{i}\cdot\exp\left(-K\cdot SP_{j}\right)\left(0.943-0.00968\cdot K\cdot SP_{j}^{0.5}\right)$ | $91.121$ |
| $C_{j}=C_{i}\cdot\exp\left(-K\cdot SP_{j}\right)\left(1.138-0.258\cdot SP_{j}^{0.5}+0.0606\cdot SP_{j}\right)$ | $91.713$ |
| $C_{j}=C_{i}\cdot\exp\left(-K\cdot SP_{j}\right)\cdot\left(1.126-0.157\cdot\left(K\cdot SP_{j}\right)^{0.5}+0.0367\cdot K^{0.5}\cdot SP_{j}\right)$ | $91.738$ |

**Table S5.** Calimera WDN - EPR results– Test B with first order data

| Expression | $R^{2}\left(\%\right)$ |
| --- | --- |
| $C_{j}=1.0068\cdot\left(K\cdot Age_{j}+C_{i}^{-1}\right)^{-1}$ | $99.758$ |
| $C_{j}=0.99678\cdot\left(K\cdot Age_{j}+C_{i}^{-1}\right)^{-1}+0.0067055\cdot C_{i}$ | $99.767$ |
| $C_{j}=0.97574\cdot\left(K\cdot Age_{j}+C_{i}^{-1}\right)^{-1}+0.025747\cdot C_{i}^{0.5}\cdot\left(K\cdot Age_{j}+C_{i}^{-1}\right)^{-0.5}$ | $99.770$ |
| $C_{j}=\left(K\cdot Age_{j}+C_{i}^{-1}\right)^{-1}\left(0.0267\left(K\cdot Age_{j}+C_{i}^{-1}\right)^{0.5}+0.942\right)$ $+0.0297C_{i}^{1.5}\left(\exp\left(-K\cdot Age_{j}\right)\right)^{0.5}$   | $99.771$ |
| $C_{j}=0.980\left(K\cdot Age_{j}+C_{i}^{-1}\right)^{-1}+0.0146C_{i}^{1.5}\left(\left(K\cdot Age_{j}+C_{i}^{-1}\right)\cdot\exp\left(-K\cdot Age_{j}\right)\right)^{0.5}$ $+0.00516C_{i}^{0.5}$   | $99.771$ |
| $C_{j}=0.9936\left(K\cdot Age_{j}+C_{i}^{-1}\right)^{-1}+0.00122\cdot C_{i}^{1.5}\cdot Age_{j}^{0.5}\cdot K\cdot\left(\exp\left(-K\cdot Age_{j}\right)\right)^{0.5}$ $+0.00465\cdot C_{i}$   | $99.771$ |
| $C_{j}=0.991\left(K\cdot Age_{j}+C_{i}^{-1}\right)^{-1}+0.00215\cdot K\cdot Age_{j}^{0.5}\cdot C_{i}^{1.5}\left(\left(K\cdot Age_{j}+C_{i}^{-1}\right)\cdot\exp\left(-K\cdot Age_{j}\right)\right)^{0.5}$ $+0.0047\cdot C_{i}^{0.5}$   | $99.772$ |

**Table S6.** Calimera WDN - EPR results– Test A with second order data

| xpression | $R^{2}\left(\%\right)$ |
| --- | --- |
| $C_{j}=0.94307\cdot\left(K\cdot SP_{j}+C_{i}^{-1}\right)^{-1}$ | $93.938$ |
| $C_{j}=1.0326\cdot\left(K\cdot SP_{j}+C_{i}^{-1}\right)^{-1}-0.10412\cdot C_{i}\cdot\exp\left(-K\cdot SP_{j}\right)$ | $94.112$ |
| $C_{j}=1.0339\cdot\left(K\cdot SP_{j}+C_{i}^{-1}\right)^{-1}-0.0676\cdot K^{0.5}\cdot C_{i}\cdot\exp\left(-K\cdot SP_{j}\right)$ | $94.143$ |
| $C_{j}=\left(K\cdot SP_{j}+C_{i}^{-1}\right)^{-1}\left(1.0117-0.0727\cdot SP_{j}^{0.5}\cdot C_{i}\cdot\exp\left(-K\cdot SP_{j}\right)\right)$ | $94.220$ |
| $C_{j}=1.1054\cdot\left(K\cdot SP_{j}+C_{i}^{-1}\right)^{-1}-C_{i}\cdot\exp\left(-K\cdot SP_{j}\right)\cdot\left(0.126+0.0351\cdot SP_{j}^{0.5}\right)$ | $94.257$ |
| $C_{j}=\left(K\cdot SP_{j}+C_{i}^{-1}\right)^{-1}\left(1.0199-0.0334\cdot SP_{j}^{0.5}\cdot K\cdot C_{i}\cdot\exp\left(-K\cdot SP_{j}\right)\right)$ | $94.287$ |
| $C_{j}=1.054\cdot\left(K\cdot SP_{j}+C_{i}^{-1}\right)^{-1}-SP_{j}^{0.5}C_{i}\exp\left(-K\cdot SP_{j}\right)\cdot\left(0.129-0.0271SP_{j}^{0.5}\right)$ | $94.431$ |
| $C_{j}=\left(K\cdot SP_{j}+C_{i}^{-1}\right)^{-1}\left(1.049-0.0424\cdot SP_{j}^{0.5}\cdot K\cdot C_{i}^{0.5}\cdot\exp\left(-K\cdot SP_{j}\right)\right)$ | $94.369$ |
| $C_{j}=1.059\cdot\left(K\cdot SP_{j}+C_{i}^{-1}\right)^{-1}+SP_{j}^{0.5}C_{i}\exp\left(-K\cdot SP_{j}\right)\cdot\left(-0.131+0.0409\cdot SP_{j}^{0.5}K^{-0.5}\right)$ | $94.467$ |
| $C_{j}=1.059\cdot\left(K\cdot SP_{j}+C_{i}^{-1}\right)^{-1}+SP_{j}^{0.5}K^{0.5}C_{i}\exp\left(-K\cdot SP_{j}\right)\cdot\left(-0.0848+0.0174SP_{j}^{0.5}\right)$ | $94.472$ |
| $C_{j}=SP_{j}^{0.5}K^{0.5}C_{i}\exp\left(-K\cdot SP_{j}\right)\cdot\left(0.00958C_{i}^{0.5}SP_{j}^{0.5}\left(K\cdot SP_{j}+C_{i}^{-1}\right)^{0.5}-0.0679\right)$ $+1.046\cdot\left(K\cdot SP_{j}+C_{i}^{-1}\right)^{-1}$ | $94.477$ |

**Table S7.** Calimera WDN - EPR results– Test B with second order data
